# Supplementary figures and images for: Phylogenomic assessment of the role of hybridization and introgression in trait evolution
Source: PLoS Genet. 2021 Aug 18;17(8):e1009701. doi: 10.1371/journal.pgen.1009701 (PMC8405015; doi:10.1371/journal.pgen.1009701)

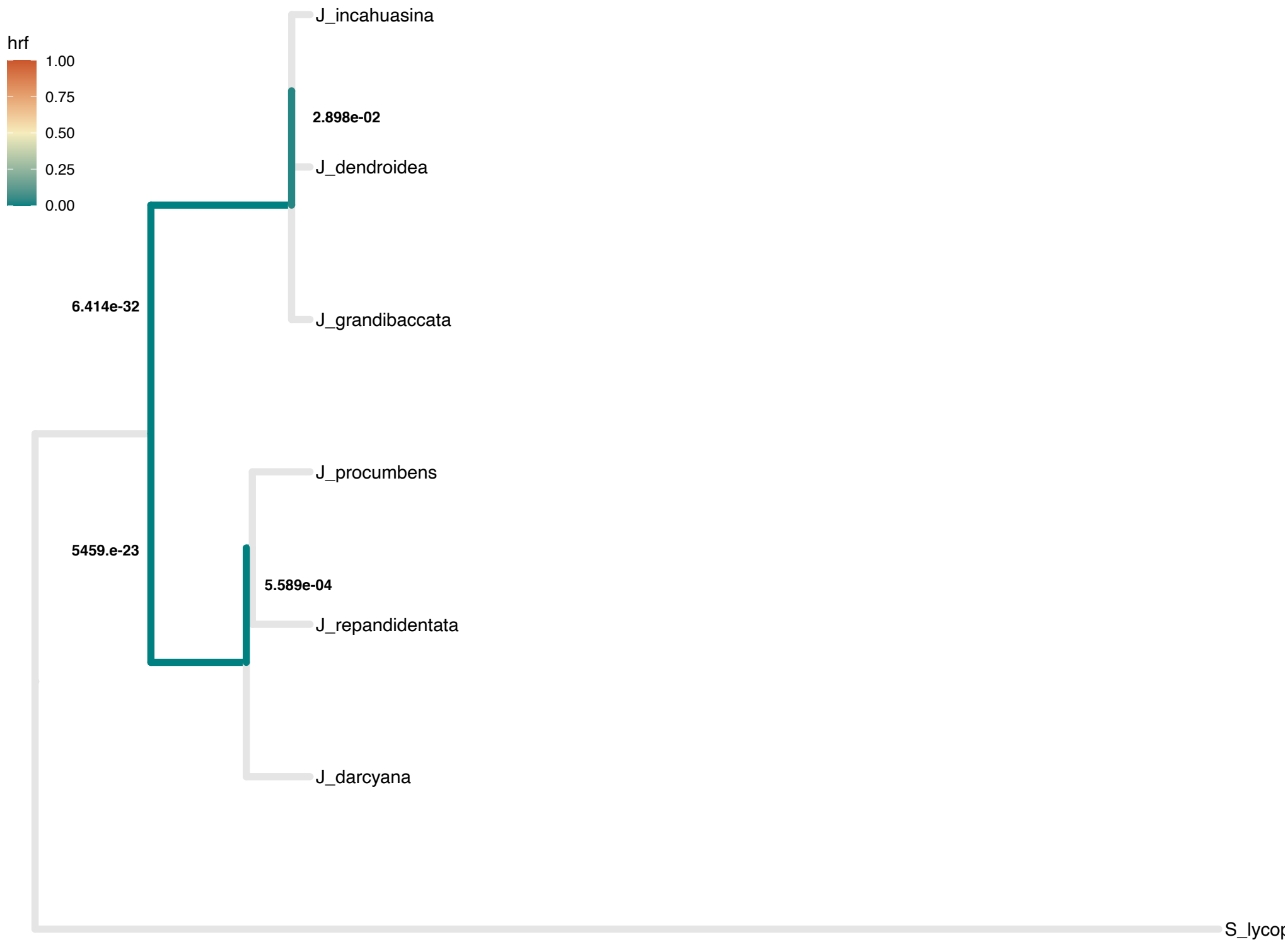

Supplement: S1 Fig — This phylogeny was derived from StarBEAST2 analysis of Jaltomata data. HRF values (branch labels and branch colors) were calculated as per Guerrero & Hahn [12]. (PDF) [file pgen.1009701.s001.pdf]

hrf

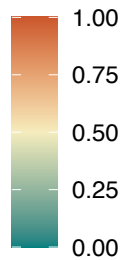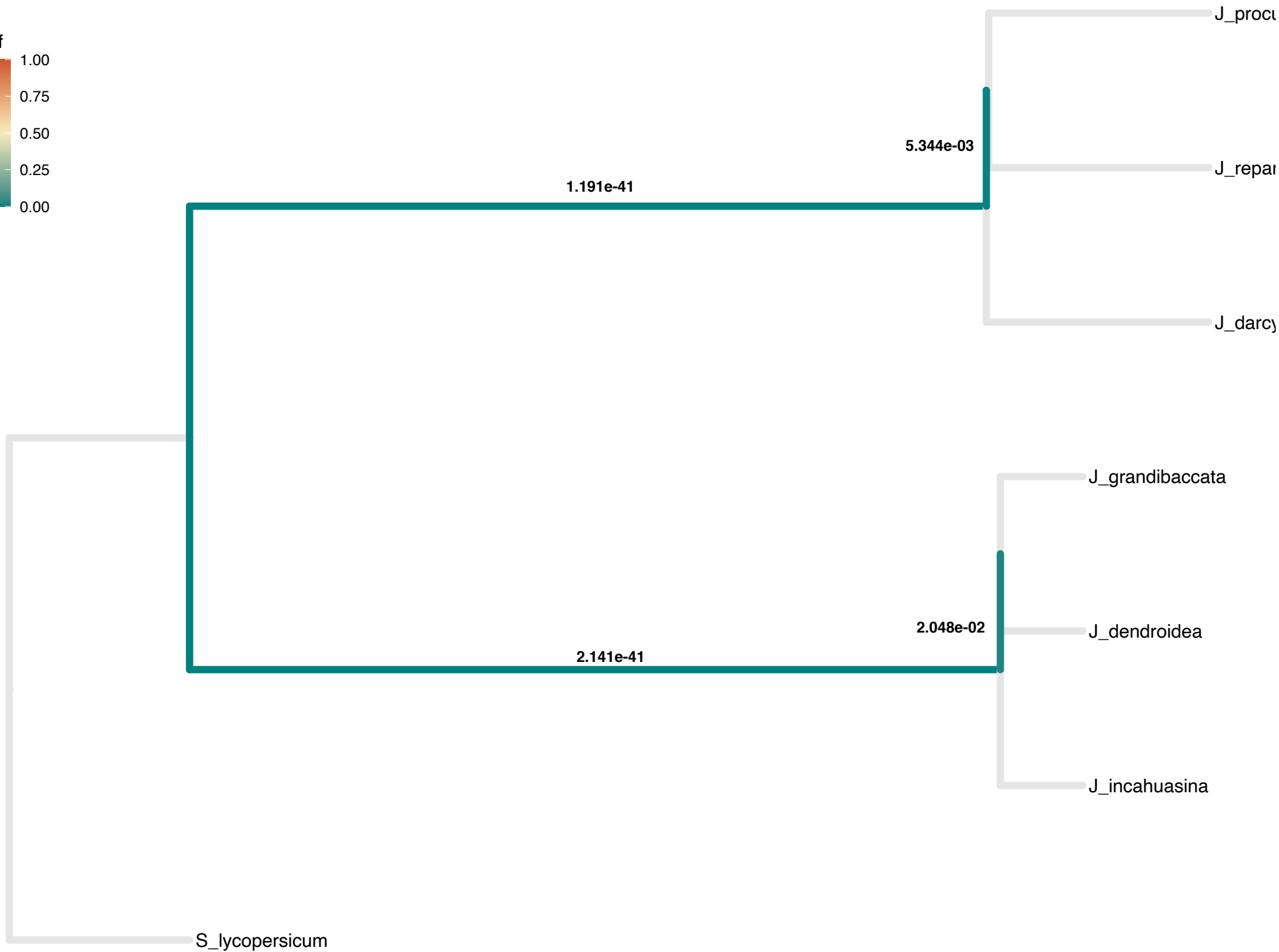

Supplement: S2 Fig — This phylogeny was derived as the major tree from MCMC_SEQ analysis of Jaltomata data. HRF values (branch labels and branch colors) were calculated as per Guerrero & Hahn [12]. (PDF) [file pgen.1009701.s002.pdf]

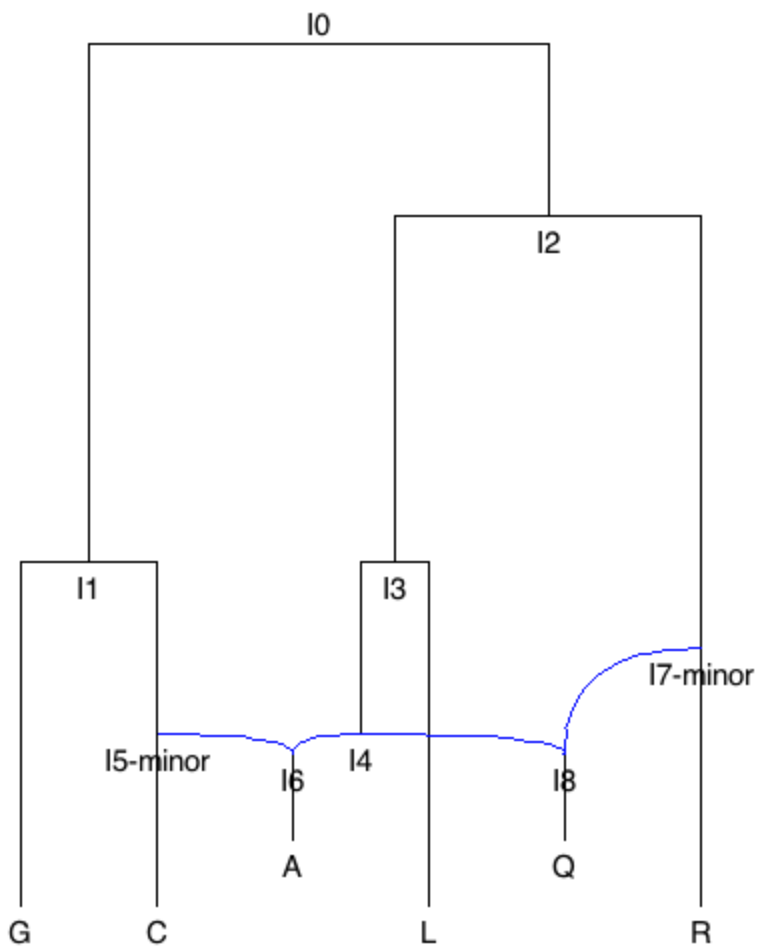

Supplement: S3 Fig — Edges leading into (from the parents) of reticulation nodes shown in blue. Minor reticulation edges indicated by internal node labels suffixed with “minor”. (PDF) [file pgen.1009701.s003.pdf]

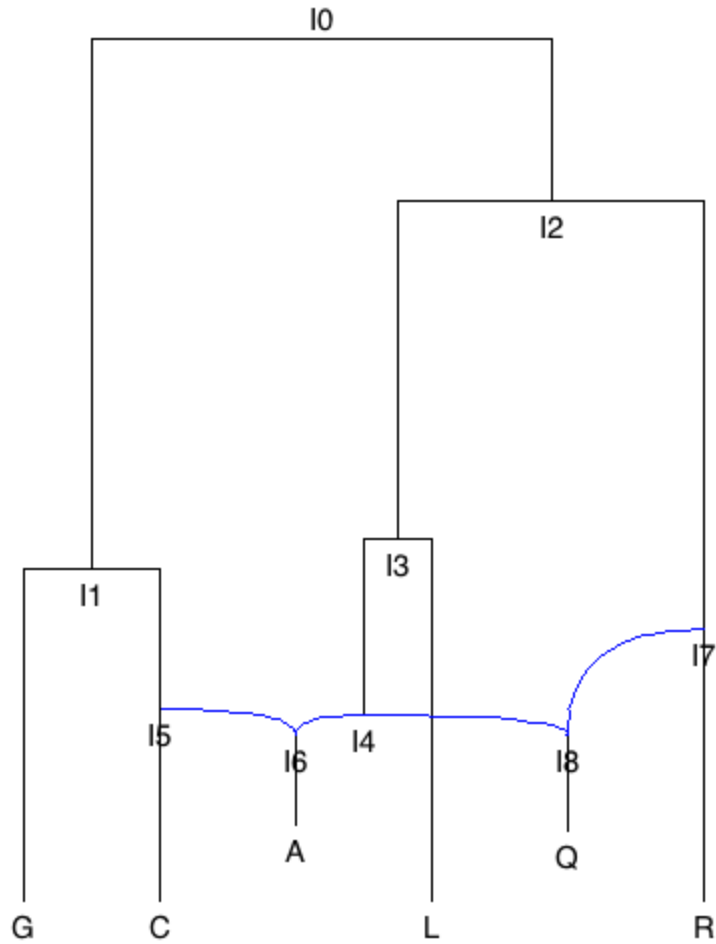

Supplement: S4 Fig — This phylogeny was estimated using MCMC_SEQ, from data simulated based on the true species network. Edges leading into reticulation nodes (from the parents to the reticulation node) shown in blue. (PDF) [file pgen.1009701.s004.pdf]

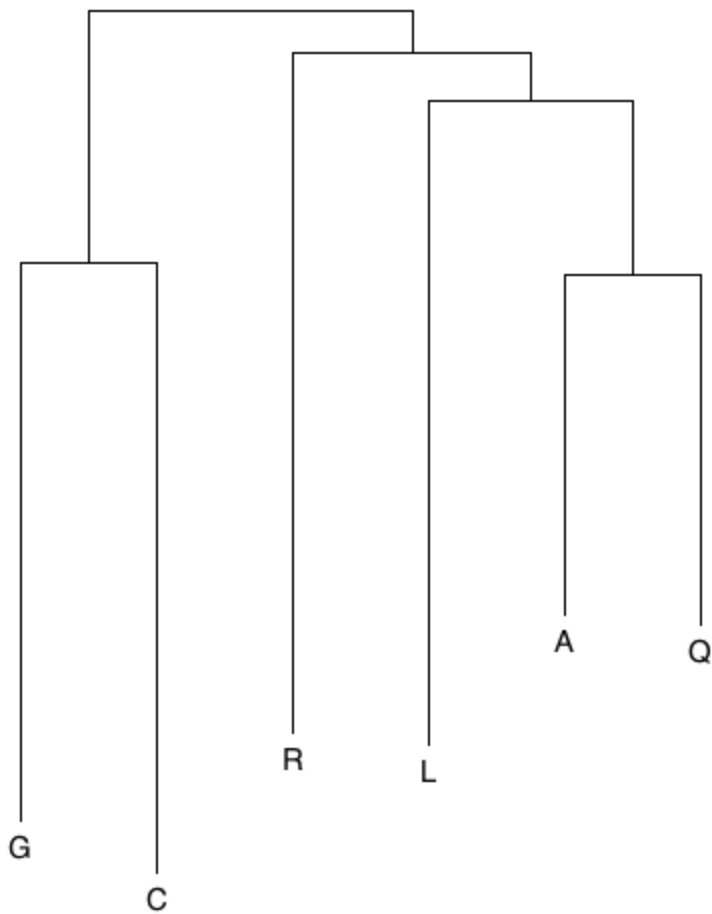

Supplement: S5 Fig — This phylogeny was estimated using StarBEAST2, from data simulated based on the true species network. (PDF) [file pgen.1009701.s005.pdf]

hrf

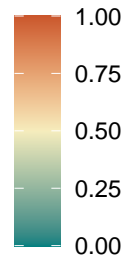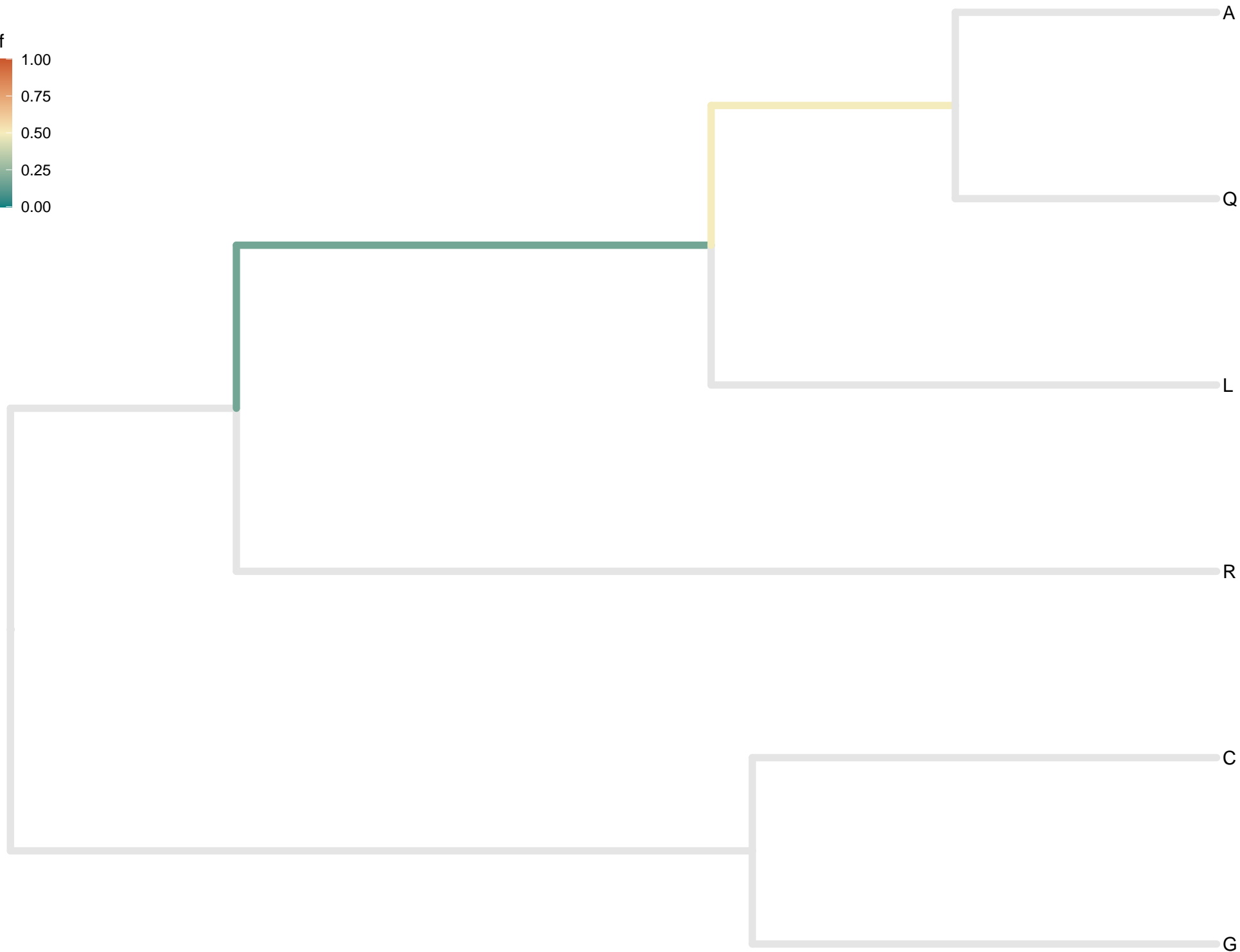

Supplement: S6 Fig — This phylogeny was derived as the major tree from MCMC_SEQ analysis of simulated data, and HRF values (branch labels and branch colors) were calculated as per Guerrero & Hahn [12]. (PDF) [file pgen.1009701.s006.pdf]

hrf

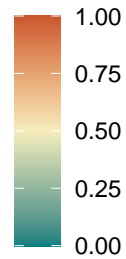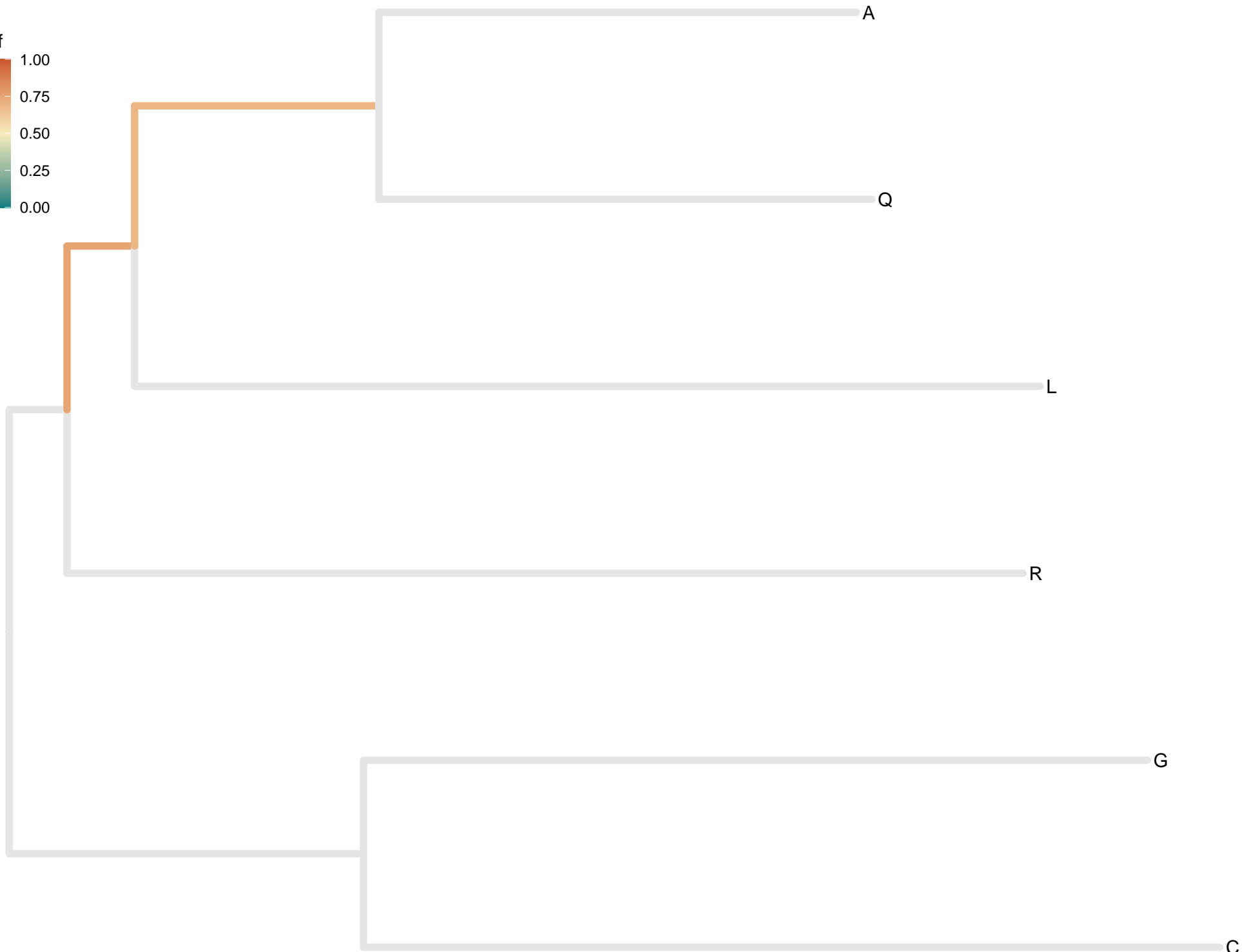

Supplement: S7 Fig — This phylogeny was derived from StarBEAST2 analysis of simulated data, and HRF values (branch labels and branch colors) were calculated as per Guerrero & Hahn [12]. (PDF) [file pgen.1009701.s007.pdf]

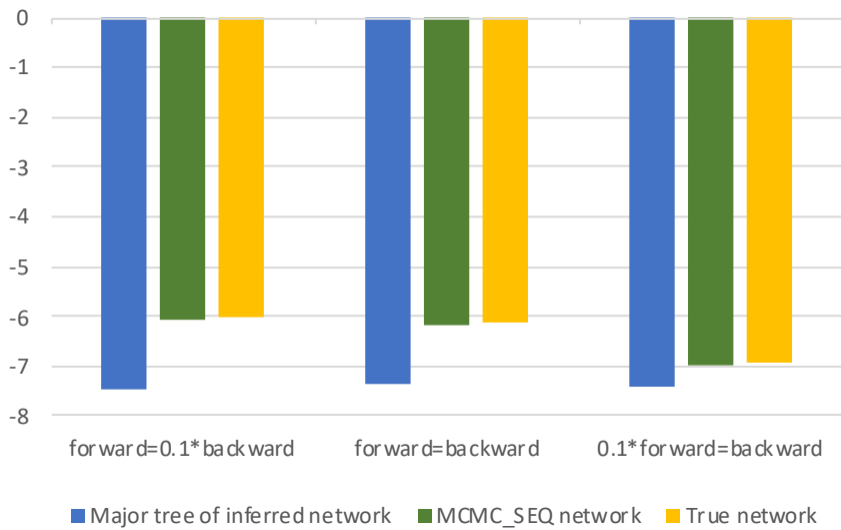

Supplement: S8 Fig — The natural logarithm of the posterior probability of the phylogenies, given the trait states of species A and C are derived and all others ancestral. (PDF) [file pgen.1009701.s008.pdf]

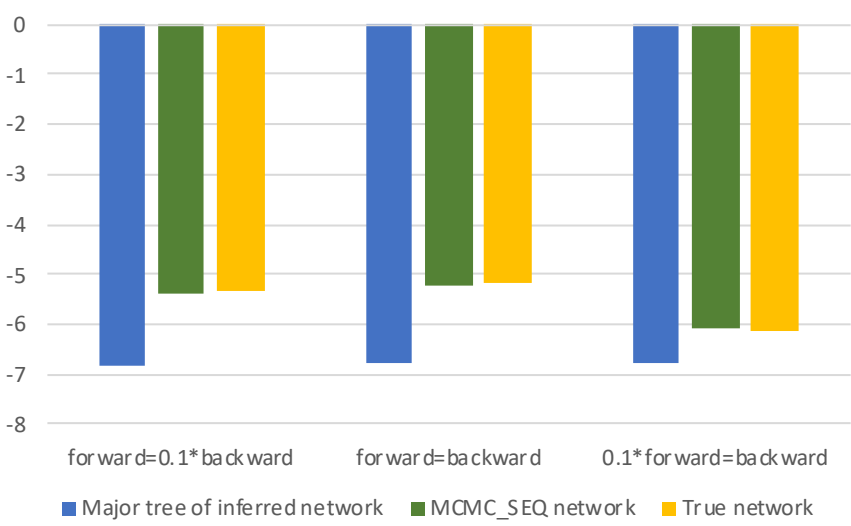

Supplement: S9 Fig — The natural logarithm of the posterior probability of the phylogenies, given the trait states of species Q and R are derived and all others ancestral. (PDF) [file pgen.1009701.s009.pdf]
